# Transcriptomic Analysis of Rat Cerebral Cortex Reveals the Potential Mechanism of Electroacupuncture Opening Blood Brain Barrier

**DOI:** 10.3389/fnins.2022.834683

**Published:** 2022-02-24

**Authors:** Congcong Ma, Lin Gan, Hao Wang, Li Ren, Yubo Lin, Yibin Zhao, Shanshan Zhang, Peng Gong, Xianming Lin

**Affiliations:** Key Laboratory of Acupuncture and Neurology of Zhejiang Province, The Third Affiliated Hospital of Zhejiang Chinese Medical University, Zhejiang Chinese Medical University, Hangzhou, China

**Keywords:** electroacupuncture, BBB, transcriptomic, mechanism, bioinformatics

## Abstract

Therapeutic treatment options for central nervous system (CNS) diseases are greatly limited by the blood-brain barrier (BBB). Electroacupuncture (EA) can be used to induce an increase in BBB permeability on rats, providing a potential approach for the delivery of drugs from the systemic circulation into the brain. However, there remains a large gap in our knowledge regarding the impact of EA on brain gene expression. This work is focused on investigating the transcriptional changes of rat cerebral cortex following EA and expression changes in genes and bioinformatic analysis was performed. We found that the potential mechanism of EA opening BBB involves receptor-mediated/carrier-mediated endocytosis (RMT/CMT), and related genes include solute carrier (SLC) transporter genes and ATP-binding cassette (ABC) transporter genes. The results also suggested that EA may affect the expression of tight junction (TJ) proteins in endothelial cells by affecting integrin binding, autophagy pathway and calcium signaling pathway, thus further affecting the permeability of blood-brain barrier. Our results provide a valuable resource that will guide mechanism research of EA opening BBB and other ways to mediate drug delivery into the brain.

## Introduction

Vascular endothelial cells in the brain are closely linked to each other through various junction proteins, and interact with pericytes and astrocytes to form a special blood-brain barrier (BBB) barrier system. BBB strictly limits the entry of neurotoxic substances, inflammatory factors and immune cells in the blood into central nervous system (CNS), and excludes the metabolites and neurotoxic substances in CNS from the brain. However, BBB also prevents certain drugs and large-molecule therapeutics from entering the brain (Banks, 2016). Clearly, higher levels of therapeutic drugs in the brain will be beneficial, reducing dose requirements and providing greater therapeutic indices. Reflecting this demand, many brain delivery technologies have been developed and tested in laboratory and clinical studies, comprising invasive technologies such as direct brain injection, intrathecal brain deliver, intracerebral grafts and deep brain stimulation and non-invasive technologies including nanoparticulate systems, focused ultrasound, biological mechanisms and intranasal brain delivery (Terstappen et al., 2021). A number of factors contribute to the physical barrier of BBB and the major are endothelial transport systems and cellular junction molecules.

Our previous study found that 2/100 Hz Electroacupuncture (EA) stimulation increased BBB permeability, reduced ZO-1 and occludin levels, and induced ultrastructural changes in TJ morphology (Zhang et al., 2020), but the molecular and signaling mechanisms of BBB opening mediated by EA are still lacking. Therefore, in this manuscript, transcriptome sequencing combined with bioinformatics analysis was used to explore the effect of specific stimulation mode electroacupuncture on the transcriptome of rat cerebral cortex, focusing on the molecules and signaling pathways related to the blood-brain barrier, in order to reveal the potential mechanism of electroacupuncture opening the blood-brain barrier, and provide potential targets and new research ideas for mediating central nervous system therapeutic drugs into the brain.

## Materials and Methods

### Experimental Animals

Adult male Sprague-Dawley rats (250–300 g) were purchased from Shanghai Laboratory Animal Center, Chinese Academy of Sciences and raised in the Laboratory Animal Center of Zhejiang Chinese Medical University. Rats were housed five per cage with *ad libitum* access to food and water at constant temperature (25 ± 2°C) with a standard 12:12 h light-dark cycle. This study was approved by the Animal Protection and Use Committee of Zhejiang University of Traditional Chinese Medicine. All procedures were carried out in accordance with the National Institutes of Health Guide for Care and Use of Laboratory Animals.

### Electroacupuncture Stimulation

Rats in EA groups were treated with disposable sterile acupuncture needles (Beijing Zhongyan Taihe Medical Device Co., Ltd., China) at GV20 (Baihui) and GV26 (Shuigou), GV20 with a needle of 25 mm in length and 0.13 mm in diameter and GV26 of a 16 mm in length and 0.07 mm in diameter. Then the needle was stimulated with a self-made relay (mode 6 s, stop 6 s) connected to the acupuncture point stimulator (HANS-200, Nanjing Jinsheng, Ltd., China). The current intensity was 3 mA, the frequency was 2/100 Hz, and the stimulation lasted for 40 min. Control rats were treated with the same binding for 40 min.

### Extraction of Cerebral Cortex Tissue

After EA, rats were anesthetized with pentobarbital (50 mg/kg). The animals were perfused with 0.9% normal saline through the left ventricle until the colorless liquid was obtained in the right atrium, and the liver became white. The brain was cut off, and the cortex tissue was quickly stripped, cut into small pieces, and put into a 1.5 mL EP tube filled with RNA later prepared in advance, so that the sample was completely immersed in the liquid, and then stored overnight at 4°C.

### RNA-Seq Library Establishment and RNA-Seq

Total RNA was isolated and purified using TRIzol reagent (Invitrogen, Carlsbad, CA, United States) following the manufacturer’s procedure. The RNA amount and purity of each sample was quantified using NanoDrop ND-1000 (NanoDrop, Wilmington, DE, United States). The RNA integrity was assessed by Bioanalyzer 2100 (Agilent, CA, United States) with RIN number > 7.0, and confirmed by electrophoresis with denaturing agarose gel. Poly (A) RNA is purified from 1 μg total RNA using Dynabeads Oligo (dT)25-61005 (Thermo Fisher Scientific, CA, United States) using two rounds of purification. Then the poly(A) RNA was fragmented into small pieces using Magnesium RNA Fragmentation Module (NEB, cat.e6150, United States) under 94°C 5–7 min. Then the cleaved RNA fragments were reverse-transcribed to create the cDNA by SuperScript™ II Reverse Transcriptase (Invitrogen, cat. 1896649, United States), which were next used to synthesize U-labeled second-stranded DNAs with E. coli DNA polymerase I (NEB, cat.m0209, United States), RNase H (NEB, cat.m0297, United States), and dUTP Solution (Thermo Fisher Scientific, cat.R0133, United States). An A-base is then added to the blunt ends of each strand, preparing them for ligation to the indexed adapters. Each adapter contains a T-base overhang for ligating the adapter to the A-tailed fragmented DNA. Single- or dual-index adapters are ligated to the fragments, and size selection was performed with AMPureXP beads. After the heat-labile UDG enzyme (NEB, cat.m0280, United States) treatment of the U-labeled second-stranded DNAs, the ligated products are amplified with PCR by the following conditions: initial denaturation at 95°C for 3 min; 8 cycles of denaturation at 98°C for 15 s, annealing at 60°C for 15 s, and extension at 72°C for 30 s; and then final extension at 72°C for 5 min. The average insert size for the final cDNA library was 300 ± 50 bp. At last, we performed the 2 × 150bp paired-end sequencing (PE150) on an Illumina Novaseq™ 6000 (LC-Bio Technology Co., Ltd., Hangzhou, China) following the vendor’s recommended protocol.

### Data Analysis

#### Sequence and Primary Analysis

Cutadapt software (^1^ version:cutadapt-1.9) was used to remove the reads that contained adaptor contamination. After removed the low quality bases and undetermined bases, we used HISAT2 software (^2^ version:hisat2-2.0.4) to map reads to the genome. The mapped reads of each sample were assembled using StringTie (^3^ version:stringtie-1.3.4d. Linux_ x86 _64). Then, all transcriptomes from all samples were merged to reconstruct a comprehensive transcriptome using gffcompare software (^4^ version:gffcompare-0.9.8. Linux_x86_64). After the final transcriptome was generated, StringTie and ballgown^5^ were used to estimate the expression levels of all transcripts and perform expression level for mRNAs by calculating FPKM.

Primary sequencing data produced by RNA-Seq (raw reads) were subjected to quality control (QC). Specific data preprocessing steps are as follows: remove reads with adaptor; reads containing N (N means undetermined base information) with a proportion of more than 5% were removed; remove low quality reads (the base number of *Q* ≤ 10 accounts for more than 20% of the whole read). Statistics of original sequencing, effective sequencing, Q20, Q30, GC content and comprehensive evaluation. The information of total reads and mapping ratio reads were shown in Table 1.

**TABLE 1:** The primers used in qPCR.

Primers	Forward	Reverse
*Cttn*	GCAGAAGGATCGGATGGACAAGAAC	TGTACTCAGGCTCAGGCTCACTAC
*Slc9a3r2*	CATGCCGAAGTTGTTGCCAGAATC	CACTGCCATCCTCATTGTCCTTCTC
*Slc38a3*	ATGGTGGTGGTGGAGGAGAAGTC	ATAGAAGGTGAGGTAGCCGAAGAGG
*Slc7a1*	CCGTATCCGCTGCTCCATTGAAC	GACCCTCTGTGAACCTTAAACCCATC
*Pigg*	GCGTAGCGATTGAGGAGTCAGTTC	ATGACATCAACAAAGCCAGGGAGAC
*Itpr3*	CATTGGTGCGTCTGGAGGAACTG	CTCGGTCTTGGTGATGTGCTTCTC
*Ltbp3*	AGGGCTACACTCAAGACAACAACATC	CTCATCCAAGCACTCATCCACATCC
*Dnaja1*	CAACCGAACCATAGTCATCACCTCTC	GCTCATCATCCTCATACGCTTCTCC
*Cblb*	ACAGACGCCACGATTTGCCTTC	GACCATTATCACAAGACCGAACAGGAG
*Hspa1b*	AAGATCACCATCACCAACGACAAGG	CCTCTTTCTCAGCCAGCGTGTTAG

#### Cluster Analysis and Screening of Differentially Expressed Genes

Clustering is calculated by using hclust function in stats package in R language, z-score normalization is performed by using scale function, and visualization is performed by using pheatmap package (Murtagh and Legendre, 2014). The differentially expressed genes (DEGs) were selected with fold change = 1.2 and *q*-Value < 0.05 by R package DESeq2^6^.

#### Gene Ontology and Kyoto Encyclopedia of Genes and Genomes Analysis

Gene ontology and Kyoto Encyclopedia of Genes and Genomes (KEGG) enrichment to the DEGs was performed using the OmicStudio tools at https://www.omicstudio.cn/tool. GO enrichment analysis can predict the functional role of DEGs from three aspects: biological process (BP), cellular component (CC) and molecular function (MF), and KEGG analysis can determine the related pathways of these genes. Next, DEGs were imported into Metascape^7^ for a network of enrichment terms (Zhou et al., 2019).

#### Gene Set Enrichment Analysis

Considering the interaction between genes, we performed GSEA enrichment analysis to connect EA intervention with changes in biological function by comparing genes with predefined gene sets in MSigDB, analyzing gene expression data, and obtains whether expression is enriched in some function (Subramanian et al., 2005). The selected gene set is c2. cp. kegg. v7.4 in MSigDB, screening with NES > 1, *P* < 0.05 and FDR < 0.25. Clusterprofiler package in R language was used for analysis and enrichplot package for visualization (Yu et al., 2012).

#### Protein–Protein Interaction Network Analysis

Interactions between proteins are central to all biological functions, and protein-protein interaction networks (PPINs) provide further insights into the protein function. Protein–protein interactions (PPIs) are experimentally detected at a large scale and used to determine which proteins in a network directly interact. The Search Tool for the Retrieval of Interacting Genes/Proteins (STRING) database^8^ is used to provide information about protein prediction and experimental interaction by setting the combination score > 0.4 as the reliability threshold, and the cytoscape software were used to construct protein–protein interaction networks and screen the hub genes using cytohubba (Shannon et al., 2003; Chin et al., 2014). Functional modules within the network were identified with the MCODE app (Bader and Hogue, 2003).

### Real-Time Quantitative PCR Analysis

According to the manufacturer’s procedure, total RNA was isolated and purified using TRIzol reagent (Invitrogen, Carlsbad, CA, United States). The primer sequence is listed in Table 1, the total RNA extracted was reversely transcribed into cDNA, and β-actin was used as an internal reference gene, using random hexamer primers (Takara bioInc., Shiga, Japan). In accordance with instructions, through cfx96 real-time system (bio rad laboratory Co., Ltd., Hercules, CA, United States), fast start universal SYBR Green master kit (Takara bioInc., China) and 25 μL reaction system for qPCR. Each reaction was performed three times and normalized to β- Actin gene expression. Cfx96 real-time system software is used to determine the CT value of each well and calculate the average value of three times. Relative quantification was determined by ΔΔCT method (Livak and Schmittgen, 2001).

### Statistical Analysis

Data in graphs are expressed as means ± SEM. Student’s *t*-test was used for comparisons between two groups. Comparison is considered significantly different if the *P*-value < 0.05.

## Results

### RNA-Seq of Transcriptome

In order to explore the possible genes involved in the mechanism of EA opening blood brain barrier, we collected the cerebral cortex of rats in EA group and control group, and analyzed the gene expression profile by RNA-Seq. The Q20% of each RNA-Seq sample reached 99.0% (Table 2), and more than 90% of the base reached mass fraction ≥ Q30.

**TABLE 2:** The information of raw reads and valid mapping ratio for electroacupuncture and control groups in RNA-Seq.

Sample	Raw read	Valid read	Valid base	Valid ratio	Q20%	Q30%
control_1	50386002	49081690	7.36G	97.41	99.97	99.05
control_2	50532582	49165486	7.37G	97.29	99.96	99.04
control_3	46976536	45706082	6.86G	97.30	99.96	99.00
control_4	46213950	45019482	6.75G	97.42	99.96	98.98
control_5	51654782	50154864	7.52G	97.10	99.96	99.00
EA_1	47846688	46200880	6.93G	96.56	99.96	99.06
EA_2	53084710	51424978	7.71G	96.87	99.97	99.08
EA_3	40298052	39082794	5.86G	96.98	99.97	99.18
EA_4	47508576	46006426	6.90G	96.84	99.97	99.05
EA_5	50579690	49280184	7.39G	97.43	99.97	99.05

### Identification of Differentially Expressed Genes

**FIGURE 1:**
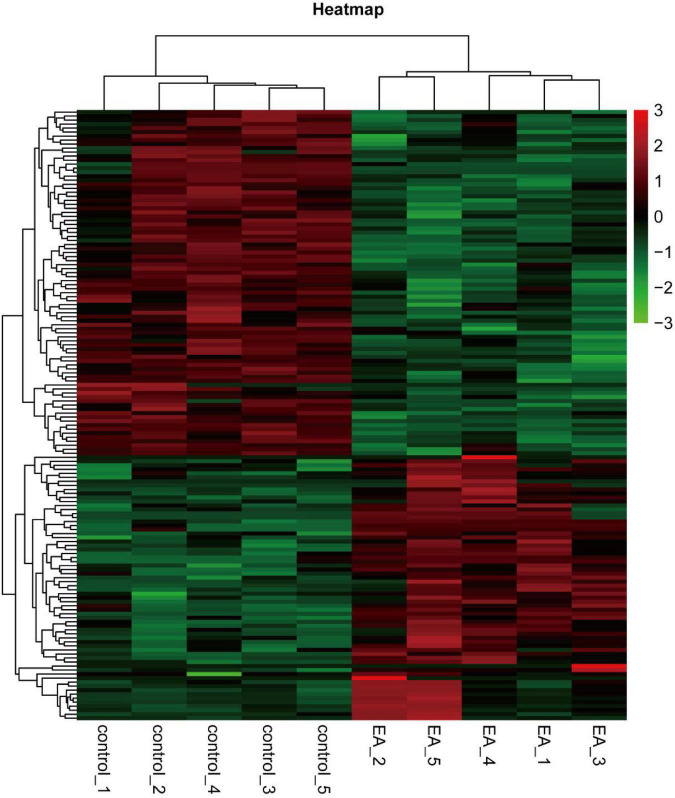
Cluster heatmap of differentially expressed genes.

**FIGURE 2:**
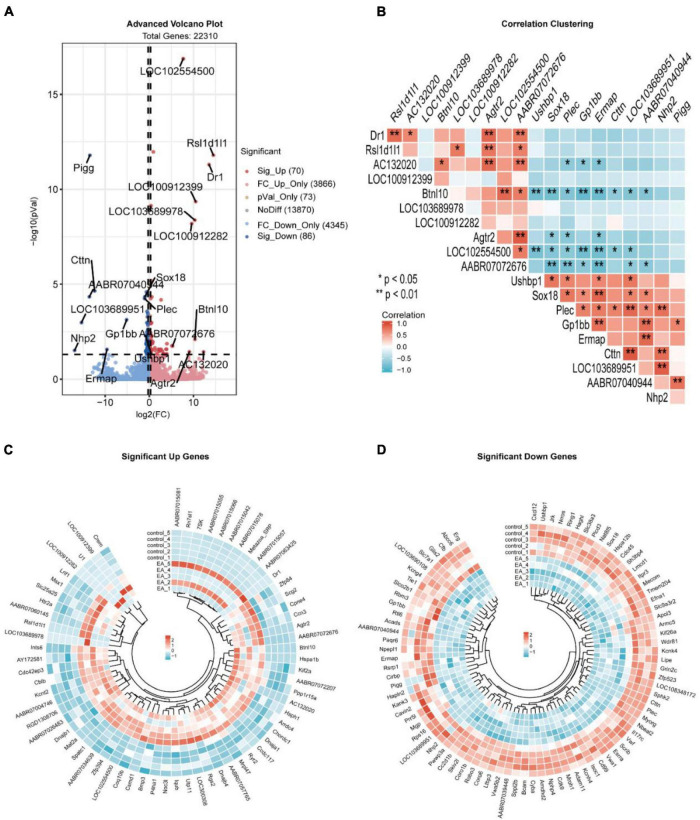
Volcano plot of DEGs **(A)**, the correlation of the first 10 significantly up-regulated genes and significantly down-regulated genes **(B)**, the expression of up-regulated genes in electroacupuncture group and control group **(C)**, and the expression of down-regulated genes in electroacupuncture group and control group **(D)**.

The 22310 genes were successfully mapped and identified from RNA-Seq. Then, we began to identify differentially expressed genes (DEGs) by FC > 1.2 and *q* < 0.05 and cluster the differentially expressed genes. The heat map showed that the differentially expressed genes were significantly separated between the EA group and the control group (Figure 1). According to this standard, we identified a total of 156 DEGs (including 70 up-regulated and 86 down regulated), which were displayed in the volcanic map, marked the most significantly up-regulated 10 genes and the most significantly down regulated 10 genes (Figure 2A), and analyzed the correlation of these genes (Figure 2B). In addition, we described the expression of significantly up-regulated and down-regulated genes in the two groups (Figures 2C,D).

The blood-brain barrier is not a single entity, but a series of characteristics, which together allow the endothelial cells of the central nervous system to strictly regulate the movement of ions, molecules and cells between blood and nerve tissue. Central nervous system endothelial cells are connected together by tight junction (TJ) to form a high resistance paracellular barrier (Furuse, 2010). Compared with peripheral endothelial cells, central nervous system endothelial cells show a lower cell transport rate (Hallmann et al., 1995). The expression of leukocyte adhesion molecules (LAMS) in endothelial cells of the central nervous system is also low, which limits the immune monitoring of the central nervous system (Engelhardt, 2008). Central nervous system endothelial cells express a variety of transporters, including a large number of special SLC transporters and ABC transporters. Genes related to blood-brain barrier were divided into groups for different BBB properties: tight junction integrity, SLC transporters, ABC transporters, LAMs, other transporters, transcytosis, endocytosis, angiogenesis or other BBB-enriched (Table 3). Our results showed that among these genes, the genes significantly up-regulated were SLC transporters related gene *Slc25a25*, endocytosis related genes *Ccdc117*, *Cdc42ep3*, *Cblb*, and *Hspa1b*, and the genes significantly down regulated were Slc transporter related genes *Slc7a1*, *Slc9a3r2*, *Slc38a3*, *Slco2b1*, ABC transporter related gene *Abcc6*, transcytosis related gene *Cavin2*, and endocytosis related genes *Cttn*, *Cdc45*, *Hspa12b.*

**TABLE 3:** Genes related to blood-brain barrier.

BBB properties	*Gene symbol*	Log2 FC (EA/control)	Adjusted *P*-value
Tight junction integrity	*Cgn*	0.02	0.982
	*Cgnl1*	–0.09	0.954
	*Cldn5*	–11.59	0.306
	*Ctnna1*	–0.09	0.869
	*Ctnna2*	0.01	0.925
	*Ctnnb1*	–0.12	0.660
	*Jaml*	0.22	0.949
	*Jam2*	0.00	0.980
	*Jam3*	–0.04	0.978
	*Ocln*	–0.47	0.236
	*Tjp1*	–0.05	0.952
	*Tjp2*	–0.08	0.928
	*Tjp3*	0.25	0.889
	*Marveld1*	–0.21	0.830
	*Marveld2*	–0.49	0.715
	*Marveld3*	5.93	0.935
	*Lsr*	–0.34	0.268
	*F11r*	–0.12	0.899
	*Emp1*	–0.01	0.996
	*Cgnl1*	–0.09	0.954
	*Pmp22*	0.02	0.955
Slc transporters	***Slc7a1***	–**0.80**	**0.033**
	***Slc9a3r2***	–0.44	**0.022**
	***Slc25a25***	**0.31**	**0.013**
	***Slc38a3***	–**0.29**	**0.002**
	***Slco2b1***	–**0.29**	**0.010**
ABC transporters	*Abca2*	–0.23	0.022
	*Abcb4*	–0.24	0.116
	*Abcc3*	–0.57	0.148
	***Abcc6***	–**0.66**	**0.015**
	*Abcd4*	–0.14	0.924
	*Abcg2*	–0.25	0.658
LAMs	*Sele*	–1.26	0.836
	*Selp*	0.32	0.979
	*Icam1*	–0.20	0.877
	*Vcam1*	0.00	0.980
	*Alcam*	0.07	0.594
	*Mcam*	–0.07	0.962
	*Ninj1*	–0.05	0.980
Other transporters	*Lrp1*	–0.18	0.188
	*Lrp10*	–0.09	0.926
	*Mfsd2a*	–0.38	0.286
	*Mfsd2b*	–8.26	0.777
Transcytosis	*Tfrc*	0.10	0.602
	*Cav1*	–0.03	0.998
	*Cav2*	0.06	0.936
	*Cav3*	–8.05	0.835
	*Cavin1*	–0.42	0.659
	***Cavin2***	–**0.62**	**0.035**
	*Cavin3*	–0.25	0.381
	*Cavin4*	–0.41	0.863
Endocytosis	*Arf1*	–0.02	0.989
	*Arf2*	0.06	0.863
	*Arf4*	0.13	0.349
	*Arf5*	–0.07	0.935
	***Cttn***	–**12.38**	**0.000**
	***Ccdc117***	**0.42**	**0.005**
	***Cdc42ep3***	**0.37**	**0.008**
	***Cdc45***	–**0.37**	**0.033**
	***Cblb***	**0.74**	**0.013**
	*Dnm1*	–0.03	0.992
	*Eps8*	–0.17	0.374
	*Eps15*	–0.02	0.992
	*Flot1*	–0.12	0.879
	*Flot2*	0.29	0.101
	*Fyn*	0.12	0.352
	***Hspa1b***	**2.67**	**0.000**
	***Hspa12b***	–**0.64**	**0.041**
Angiogenesis	*Angpt4*	0.37	0.879
	*Angpt1*	0.03	0.980
	*Angpt2*	–0.10	0.970
	*Egr1*	0.03	0.961
	*Egr2*	0.13	0.861
	*Egr3*	0.03	0.935
	*Egr4*	–0.16	0.886
	*Fgfr1*	–0.13	0.507
	*Fgfr2*	–0.04	0.995
	*Itgb1*	0.03	0.922
	*Itgb2*	0.04	0.954
	*Itgb3*	–0.29	0.935
	*Lgals1*	–0.10	0.961
	*Lgals2*	–7.70	0.935
	*Lgals3*	–0.14	0.968
	*Mmp9*	–0.08	0.980
	*Pdgfa*	–0.07	0.953
	*Pdgfb*	–0.05	0.980
	*Tgfb2*	0.14	0.692
	*Vegfa*	–0.13	0.949
	*Vegfb*	–0.31	0.605

*Genes in bold refer to differentially expressed genes (DEGs) identified by FC > 1.2 and q < 0.05.*

### Differential Expression Detected by Transcriptome Was Strongly Related to qPCR Results

**FIGURE 3:**
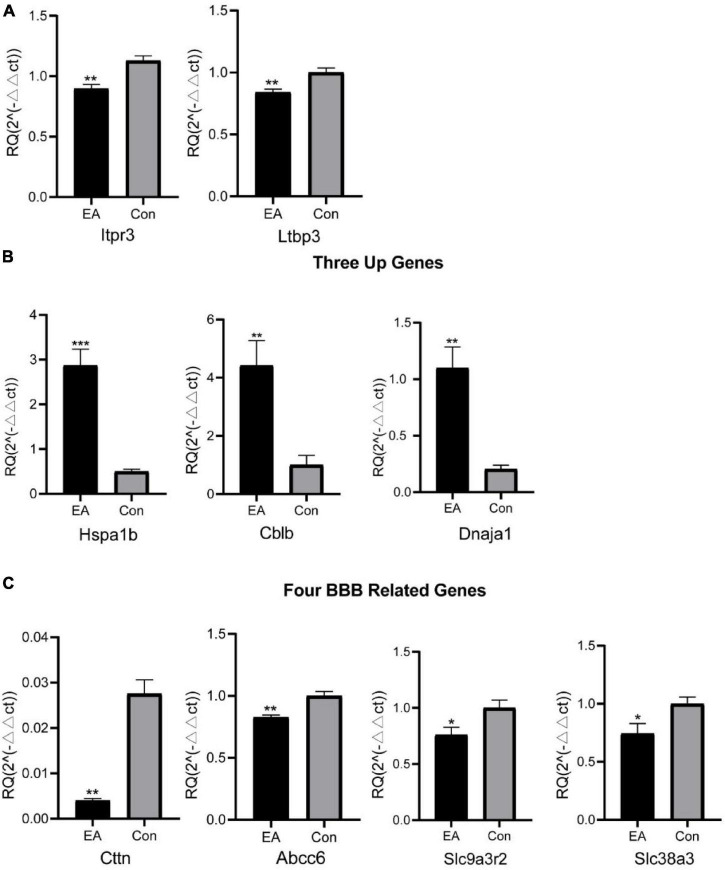
RNA-Seq results were validated by qPCR. Two randomly selected down-regulated DEG expression **(A)** from RNA-Seq and three up-regulated DEG **(B)** expression were detected by qPCR, and **(C)** four typical genes related to BBB. *p < 0.05, **p < 0.01, and ***p < 0.00. Student’s *t*-test was used for comparisons.

Next, we set to examine the reliability of our RNA-Seq data using qPCR. We randomly selected three up-regulated genes (*Hspa1b, Cblb, Dnaja1*), two down-regulated genes (*Itpr3, Ltbp3*) and four genes related to BBB (*Cttn, Abcc6, Slc9a3r2, Slc38a3*) for qPCR verification. The results of qPCR showed that the expression of *Hspa1b, Cblb* and *Dnaja1* were all significantly up-regulated (Figure 3B), whereas *Itpr3* and *Ltbp3* were significantly downregulated in EA rats vs. control rats (Figure 3A), which are consistent with RNA-Seq data. Furthermore, the qPCR results of the four selected genes of interest were also consistent with the sequencing results (Figure 3C). Thus, qPCR results provide evidence that RNA-Seq data for gene expression profiling are reliable.

### Gene Ontology and Kyoto Encyclopedia of Genes and Genomes Analysis of Differentially Expressed Genes

**FIGURE 4:**
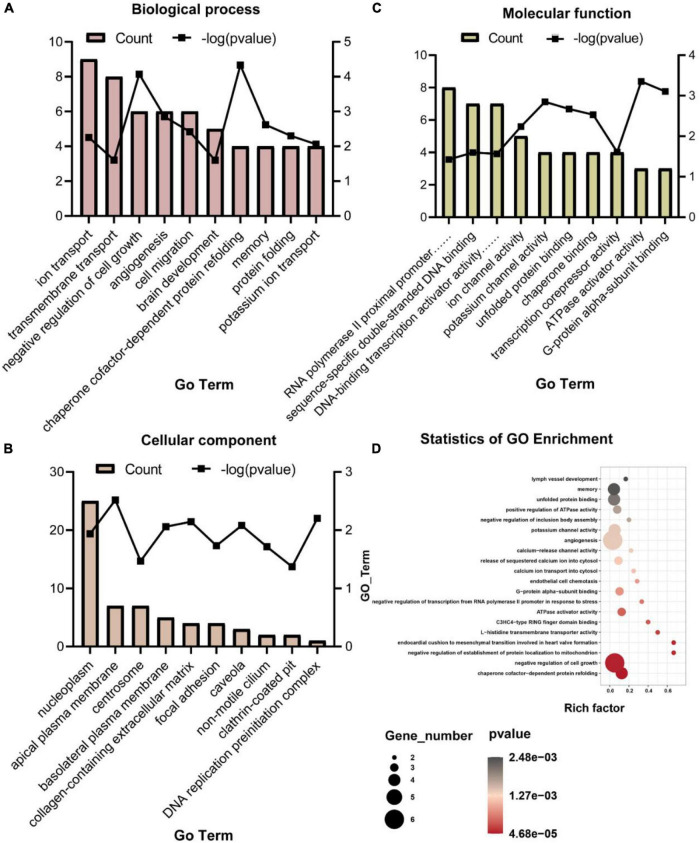
Gene ontology items with top 10 counts and significant enrichment (*P* < 0.05) in BP **(A)**, CC **(B)**, and MF **(C)**, and the statistics of the top 20 terms with the most significant enrichment **(D)**.

To analyze molecular mechanisms and signaling pathways further, we performed GO Term and KEGG pathway enrichment analysis on DEGs. As shown in Figure 4, we show the GO items with top 10 counts and significant enrichment (*P* < 0.05) in biological processes (BP), cell composition (CC) and molecular function (MF), respectively (Figures 4A-C). Among them, the BP that may be related to the composition of BBB and material transport are ion transport (GO:0006811), transmembrane transport (GO:0055085), angiogenesis (GO:0001525), brain development (GO:0007420), potassium ion transport (GO:0006813); the CC are apical plasma membrane (GO:0016324), basolateral plasma membrane (GO:0016323), collagen-containing extracellular matrix (GO:0062023), focal adhesion (GO:0005925), caveola (GO:0005901), clathrin-coated pit (GO:0005905); the MF are ion channel activity (GO:0005216), potassium channel activity (GO:0005267). Then we list the statistics of the top 20 terms with the most significant enrichment (minimum *p-*Value) (Figure 4D). Among these GO terms, what may be related to BBB are potassium channel activity (GO:0005267), angiogenesis (GO:0001525), calcium-release channel activity (GO:0015278), release of sequestered calcium ion into cytosol (GO:0051209), calcium ion transport into cytosol (GO:0060402), endothelial cell chemotaxis (GO:0035767), L-histidine transmembrane transporter activity (GO:0005290). All GO terms associated with the BBB are listed in Table 4.

**TABLE 4:** Details of all gene ontology terms related to blood-brain barrier.

GO function	GO ID	GO term	DEGs	*P*-value
BP	GO:0006811	ion transport	*Kcnt2, Ryr2, Kcng4, Kcnh4, Slco2b1, Kcnk4, Itpr3, Grin2c, Slc38a3*	0.0056
	GO:0055085	transmembrane transport	*Abcc6, Itpr3, Ryr2, Slco2b1, Kcnh4, Kcng4, Slc25a25, Slc7a1*	0.0248
	GO:0001525	angiogenesis	*Plcd3, Scg2, Sox18, Efna1, Ccn3, Tie1*	0.0014
	GO:0007420	brain development	*Rgs2, Cttn, Cxcl12, Slc38a3, Sphk2*	0.0251
	GO:0006813	potassium ion transport	*Kcnt2, Kcnh4, Kcng4, Kcnk4*	0.0087
	GO:0071805	potassium ion transmembrane transport	*Kcnt2, Kcnk4, Kcnh4, Kcng4*	0.0092
	GO:0001568	blood vessel development	*Tie1, Sox18, Sphk2*	0.0080
	GO:0001938	positive regulation of endothelial cell proliferation	*Cxcl12, Scg2, Cyba*	0.0090
	GO:0070588	calcium ion transmembrane transport	*Ryr2, Slc25a25, Itpr3*	0.0353
	GO:0035767	endothelial cell chemotaxis	*Coro1b, Ccn3*	0.0008
	GO:0060402	calcium ion transport into cytosol	*Ryr2, Itpr3*	0.0011
	GO:0090280	positive regulation of calcium ion import	*Cxcl12, Sphk2*	0.0044
	GO:0006865	amino acid transport	*Slc7a1, Slc38a3*	0.0339
	GO:1903810	L-histidine import across plasma membrane	*Slc7a1*	0.0063
	GO:2000487	positive regulation of glutamine transport	*Slc38a3*	0.0063
	GO:0015817	histidine transport	*Slc38a3*	0.0063
	GO:0051284	positive regulation of sequestering of calcium ion	*Ryr2*	0.0125
	GO:1901726	negative regulation of histone deacetylase activity	*Sphk2*	0.0125
	GO:0006867	asparagine transport	*Slc38a3*	0.0125
	GO:1900924	negative regulation of glycine import across plasma membrane	*Rgs2*	0.0125
	GO:0060401	cytosolic calcium ion transport	*Ryr2*	0.0125
	GO:0015819	lysine transport	*Slc7a1*	0.0125
	GO:0015822	ornithine transport	*Slc7a1*	0.0187
	GO:0071603	endothelial cell-cell adhesion	*Ccn3*	0.0187
	GO:1903826	arginine transmembrane transport	*Slc7a1*	0.0187
	GO:1901896	positive regulation of ATPase-coupled calcium transmembrane transporter activity	*Ryr2*	0.0249
	GO:0030947	regulation of vascular endothelial growth factor receptor signaling pathway	*Tmem204*	0.0249
	GO:0089709	L-histidine transmembrane transport	*Slc38a3*	0.0249
	GO:0015809	arginine transport	*Slc7a1*	0.0311
	GO:0043535	regulation of blood vessel endothelial cell migration	*Efna1*	0.0311
	GO:1990822	basic amino acid transmembrane transport	*Slc7a1*	0.0311
	GO:0071896	protein localization to adherence junction	*Scrib*	0.0311
	GO:0097553	calcium ion transmembrane import into cytosol	*Grin2c*	0.0371
	GO:0015807	L-amino acid transport	*Slc7a1*	0.0371
	GO:0006868	glutamine transport	*Slc38a3*	0.0371
	GO:0015808	L-alanine transport	*Slc38a3*	0.0432
	GO:1903348	positive regulation of bicellular tight junction assembly	*Nphp4*	0.0432
	GO:0043615	astrocyte cell migration	*Scrib*	0.0432
	GO:0014808	release of sequestered calcium ion into cytosol by sarcoplasmic reticulum	*Ryr2*	0.0492
	GO:0038203	TORC2 signaling	*Prr5l*	0.0311
CC	GO:0016323	basolateral plasma membrane	*Slc38a3, Slc7a1, Abcc6, Hspa1b, Scrib*	0.0087
	GO:0062023	collagen-containing extracellular matrix	*Mgp, Vwf, Ltbp3, Ccn3*	0.0072
	GO:0005901	caveola	*Lipe, Cavin2, Htr2a*	0.0083
	GO:0005905	clathrin-coated pit	*Cttn, Sh3bp4*	0.0424
	GO:0062023	collagen-containing extracellular matrix	*Mgp, Vwf, Ltbp3, Ccn3*	0.0071
MF	GO:0005216	ion channel activity	*Itpr3, Kcnh4, Grin2c, Kcng4, Ryr2*	0.0058
	GO:0005267	potassium channel activity	*Kcnk4, Kcnh4, Kcnt2, Kcng4*	0.0014
	GO:0005290	L-histidine transmembrane transporter activity	*Slc7a1, Slc38a3*	0.0002
	GO:0015278	calcium-release channel activity	*Itpr3, Ryr2*	0.0014
	GO:0015171	amino acid transmembrane transporter activity	*Slc38a3, Slc7a1*	0.0133
	GO:0098782	mechanosensitived potassium channel activity	*Kcnk4*	0.0063
	GO:0005228	intracellular sodium activated potassium channel activity	*Kcnt2*	0.0063
	GO:0070089	chloride-activated potassium channel activity	*Kcnt2*	0.0063
	GO:0015182	L-asparagine transmembrane transporter activity	*Slc38a3*	0.0187
	GO:0005219	ryanodine-sensitive calcium-release channel activity	*Ryr2*	0.0187
	GO:0048763	calcium-induced calcium release activity	*Ryr2*	0.0249
	GO:0005220	inositol 1,4,5-trisphosphate-sensitive calcium-release channel activity	*Itpr3*	0.0249
	GO:0022849	glutamate-gated calcium ion channel activity	*Grin2c*	0.0311
	GO:0015181	arginine transmembrane transporter activity	*Slc7a1*	0.0311
	GO:0015186	L-glutamine transmembrane transporter activity	*Slc38a3*	0.0371
	GO:0015174	basic amino acid transmembrane transporter activity	*Slc7a1*	0.0371
	GO:0004972	NMDA glutamate receptor activity	*Grin2c*	0.0371
	GO:0015180	L-alanine transmembrane transporter activity	*Slc38a3*	0.0492
	GO:0015271	outward rectifier potassium channel activity	*Kcnt2*	0.0492
	GO:0005178	integrin binding	*Vwf, Ccn3, Cxcl12*	0.0426

**FIGURE 5:**
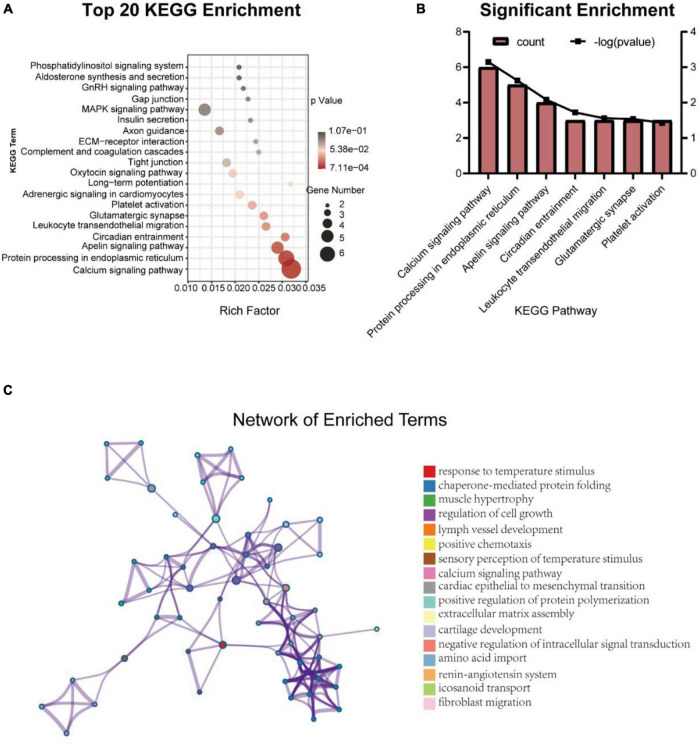
Top 20 Kyoto Encyclopedia of Genes and Genomes enrichment pathways **(A)** and the significant enrichment pathways **(B)**, and the network of enrichment terms colored by cluster ID **(C)**.

Kyoto Encyclopedia of Genes and Genomes results predicted the signal pathways related to DEGs (Figure 5A). There were 7 significantly enriched (*P* < 0.05) pathways (Figure 5B) and 15 pathways with more than or equal to 3 DEGs (Table 5). Calcium signaling pathway (4020) may be related to BBB under the intervention of EA and the DEGs involved in this signaling pathway are *Plcd3*, *Ryr2*, *Itpr3*, *Htr2a*, *Grin2c*, and *Sphk2.* In addition, apelin signaling pathway (4371) and platelet activation (4611) signaling pathway are also associated with EA intervention, involved DEGs are *Itpr3, Lipe, Ryr2, Sphk2* in the former and *Gp1bb, Vwf, Itpr3* in the later.

**TABLE 5:** Enrichment pathways with more than or equal to three differentially expressed genes.

Pathway ID	Pathway name	Genes	*P*-value
4020	Calcium signaling pathway	*Plcd3, Ryr2, Itpr3, Htr2a, Grin2c, Sphk2*	0.00
4141	Protein processing in endoplasmic reticulum	*Hsph1, Hspa1b, Dnaja1, Dnajb1, Ppp1r15a*	0.00
4371	Apelin signaling pathway	*Itpr3, Lipe, Ryr2, Sphk2*	0.01
4010	MAPK signaling pathway	*LOC100912399, Mecom, Hspa1b, Efna1*	0.09
4713	Circadian entrainment	*Ryr2, Itpr3, Grin2c*	0.02
4670	Leukocyte transendothelial migration	*Cyba, Cd99, Cxcl12*	0.03
4724	Glutamatergic synapse	*Grin2c, Itpr3, Slc38a3*	0.03
4611	Platelet activation	*Gp1bb, Vwf, Itpr3*	0.04
4261	Adrenergic signaling in cardiomyocytes	*Ryr2, Crem, Agtr2*	0.05
4921	Oxytocin signaling pathway	*Ryr2, Rgs2, Itpr3*	0.06
4530	Tight junction	*Scrib, LOC100912399, Cttn*	0.07
4360	Axon guidance	*Efna1, Cxcl12, Robo3*	0.09
4024	cAMP signaling pathway	*Grin2c, Lipe, Ryr2*	0.12
4060	Cytokine-cytokine receptor interaction	*Cxcl12, Il17rc, Bmp3*	0.20
4080	Neuroactive ligand-receptor interaction	*Htr2a, Agtr2, Grin2c*	0.33

The term with the most statistical significance in the cluster is selected as the term representing the cluster, and then a rich subset of terms is selected and displayed as a network diagram to further determine the relationship between terms (Figure 5C). Among them, calcium signaling pathway is related to the sensory perception of temperature stimulus.

### Gene Set Enrichment Analysis

Gene Set Enrichment Analysis (GSEA) revealed the enrichment of EA responsive gene sets, identifying the regulation of autophagy as the top differentially modulated pathway, display NES of 1.564 (*P* = 0.00, FDR = 0.083). Consistently, GO analysis also revealed a change in TORC2 signaling (GO:0038203, *P* = 0.03) as shown in Table 4. MTOR (the mechanism target of rapamycin kinase) signal is the most well-known autophagy regulator and MTOR signal is composed of two multi-protein complexes with different structures and functions, mTORC1 and mTORC2.

### Protein–Protein Interactions Analysis

**FIGURE 6:**
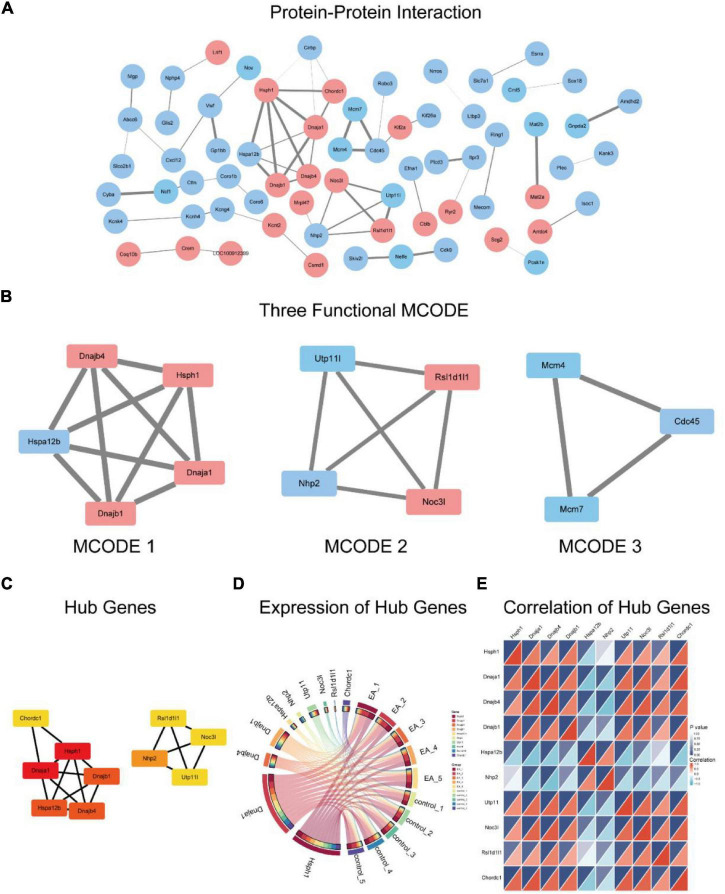
Protein-protein interaction of the DEGs **(A)**, red represents up-regulation genes, blue represents down-regulation genes, and the thicker the line, the higher the connection score. Three functional modules within the PPI **(B)**. 10 hub genes **(C)** and their expression among samples **(D)**, correlation between 10 hub genes **(E)**.

The PPI network was constructed by using DEGs (Figure 6A). The three significant modules constructed from the PPI network of the DEGs including module 1 containing 5 nodes and 10 edges (MCODE score = 5), module 2 with 4 nodes and 6 edges (MCODE score = 4), and module 3 including 3 nodes and 3 edges (MCODE score = 3) (Figure 6B). MCODE 1 combined is primarily associated with protein folding, MCODE 2 is mainly involved in rRNA pseudouridine synthesis while MCODE 3 is related to DNA replication.

The 10 hub genes screened were *Dnaja1*, *Dnajb1*, *Dnajb4*, *Hsph1*, *Chordc1, Nhp2*, *Utp11*, *Noc3l*, *Rsl1d1l1* (Figure 6C). According to the expression of hub genes in the samples (Figure 6D), it is suggested that the expression of *Hsph1* and *Dnaja1* is the highest, followed by *Dnajb1*, *Dnajb4* and *Utp11*. However, *Chordc1*, *Hspa12b*, *Nhp2*, and *Noc3l* are less expressed in the samples, while *Rsl1d1l1* is very little. Figure 6E shows the correlation of gene expression of hub genes.

## Discussion

In the development of drugs for the treatment of CNS diseases, achieving adequate BBB transmission is a key challenge. The BBB is formed by brain microvascular endothelial cells that are sealed by tight junctions, which makes it an important obstacle to most brain therapies. Therefore, poor BBB permeability is the main reason for limiting the application of newly developed CNS therapeutic drugs in clinical practice.

Multiple ABC proteins are expressed in the BBB lumen and blood-oriented inner cortical membrane, which limits the permeability of a large number of toxins, including therapeutic agents (Miller, 2015). ABC transporters are ATP-driven efflux pumps of exogenous and endogenous metabolites, and their high expression in BBB leads to drug resistance in CNS. The decreased expression and/or functional activity of ABC-BBB transporters in Alzheimer’s disease (AD) and Parkinson’s disease (PD) patients (Zlokovic, 2011), and in the brain of AD animal models proved that the decreased expression and/or functional activity of ABC-BBB transporters led to the accumulation of amyloid beta peptide (Aβ) (Cirrito et al., 2005). However, the clinical potential of ABC transporters for disease management and drug delivery improvement remains unclear. EA can reduce the expression of *Abcc6* (Table 3), suggesting that further studies can explore whether electroacupuncture can mediate the clearance of harmful sediments such as Aβ and the delivery of CNS therapeutic drugs by affecting the expression of *Abcc6*. Furthermore, EA intervention can change the expression of multiple SLC, including *Slc7a1*, *Slc9a3r2*, *Slc38a3*, *Slco2b1*, and *Slc25a25* (Table 3). The non-invasive delivery of therapeutic drugs through BBB may use endogenous processes, such as adsorption-mediated cell transport, CMT and RMT (Terstappen et al., 2021). Among them, the CMT system is expressed by genes in the SLC transporter gene family, including more than 300 transporter genes encoding membrane-binding proteins, which are helpful for the trans-membrane transport of multiple substrates (Lin et al., 2015). In BBB, SLC proteins promote cross-cellular transport of multiple molecules, including carbohydrates, amino acids, monocarboxylic acids, hormones, fatty acids, nucleotides, organic anions, amines, choline and vitamins (Zlokovic, 2008; Daneman and Prat, 2015; Pardridge, 2015). It is worth noting that *Cavin2* is also a gene related to transcytosis, which can be significantly down regulated by electroacupuncture; Other genes related to endocytosis include *Cttn*, *Cdc45*, *Hspa12b*, *Cdcc117*, *Cdc42ep3*, Cblb and *Hspa1b* (Table 3). The role of these genes in maintaining the integrity of BBB should be further studied.

Basement membrane (BM) is one of the important components of BBB because endothelial cells are connected to the BM through integrins. In addition, integrins also interact with extracellular matrix proteins such as laminin, collagen and perlecan, and mediate signal transduction by activating ECM ligands, growth factors and growth factor receptors (Baeten and Akassoglou, 2011). Our GO analysis showed that the mechanism of EA intervention involved integrin binding (GO:0005178, *P* = 0.0426) and collagen-containing extracellular matrix (GO:0062023, *P* = 0.0071) (Table 4). Mice lacking β1- integrin in endothelial cells produce abnormal cadherin signaling, claudin-5 deficiency and immature BBB (Yamamoto et al., 2015). Similarly, mice lacking laminin secreted by astrocytes showed BBB breakdown (Yao et al., 2014).

It is well known that the TJ in the BBB is a dynamic structure. TJ proteins undergo changes in expression, subcellular localization, post-translational modifications and protein-protein interactions under physiological and pathophysiological conditions (Huber et al., 2001). Interactions between TJ proteins and intracellular signaling pathways have begun to be elucidated (Bazzoni and Dejana, 2004). The cultured epithelial cells in the medium lacking Ca^2 +^ showed the loss of cell membrane ZO-1, ZO-2 and thromboxane, and the increase of related paracellular permeability (Klingler et al., 2000). Interestingly, although artificially increasing intracellular [Ca^2 +^] does not change BBB TJ (Brown et al., 2004), the treatment with Ca^2 +^ channel blocker SKF 96365 can improve the permeability increase effect of hypoxia/hyperglycemia on BBB through a mechanism that seems to involve the recruitment of occludin into the cell membrane (Brown and Davis, 2005). The results of KEGG analysis showed that the most significant enrichment pathway was the calcium signaling pathway, and the differential genes associated with this pathway are *Plcd3, Ryr2, Itpr3, Htr2a, Grin2c, Sphk2* (Figure 6 and Table 5). *Itpr3* is an inositol 1,4,5-triphosphate receptor, type 3 (InsP3R3), encoding a receptor for inositol 1,4,5-trisphosphate, a second messenger that mediates the release of intracellular calcium. Although EA intervention did not significantly change the mRNA level of TJ proteins in this experiment, the previous study of our research group found that EA intervention could affect the expression level of ZO-1 and occluding (Zhang et al., 2020). Therefore, it is not excluded that EA regulates the subcellular localization and post-translational modification of TJ proteins through the calcium signaling pathway. The next step can study the effect of EA intervention on the protein expression level of TJ proteins and the possible role of calcium signaling in it.

**FIGURE 7:**
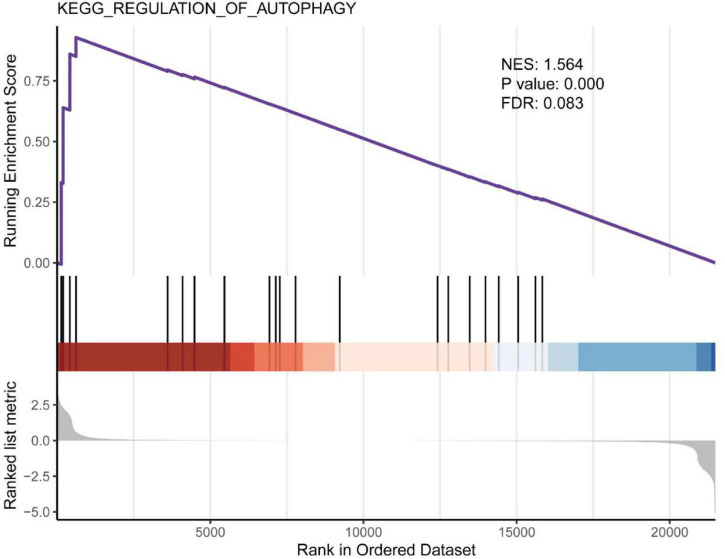
Gene set enrichment analysis for “regulation of autophagy.” Screening with NES > 1, *P* < 0.05 and FDR < 0.25.

Autophagy persists at the basic level of cells and is the mechanism of organelle or protein degradation and recycling (Lakhan et al., 2013). It is stimulated by a variety of pathological processes and participates in the elimination of organelle and protein decomposition, thereby avoiding excessive damage and cell dysfunction in various organs and cells (De Meyer et al., 2015). *In vivo* experiments showed that rapamycin (an autophagy inducer) led to the decrease of ZO-1 and the increase of BBB permeability, and confirmed the role of autophagy in regulating paracellular permeability (Zhang et al., 2018). However, studies have reported that CLDN5 (claudin 5) is abnormally accumulated in the cytoplasm of BMECs in stroke patients, accompanied by autophagy activation. Studies on zebrafish *in vivo* and *in vitro* cells showed that the BBB decomposition was caused by the redistribution of CLDN5 mediated by CAV1 (caveolin 1) into the cytoplasm under hypoxia. At the same time, autophagy is activated, which is mainly helpful degradation of CAV1 and CLDN5 in BMECs cytoplasm. These data suggest that autophagy plays a role in BBB integrity by regulating the redistribution of CLDN5 (Yang et al., 2021). Also, autophagy involves the dynamic disposition of intracellular and extracellular components, theoretically affecting BBB penetration. For example, autophagy-mediated lysosomal degradation pathway is involved in the degradation of PPR/*p* TRAIL in brain capillary endothelial cells and prevents them from penetrating BBB. The pre-inhibition of BBB autophagy by wortmannin loaded liposomes (Wtmn-Lip) can increase the accumulation of non-viral gene vector PPR in the brain without damaging the BBB tight junction (Wang et al., 2020). In the study of APP/PS1 mice, LSP (Lychee seed polyphenol) induced autophagy in bEnd.3 cells through AMPK/mTOR/ULK1 pathway and increased TJs expression (Xiong et al., 2021). The above data show that autophagy can regulate BBB permeability and material transport, but there is still no direct evidence to explain the exact relationship between the two, which may be related to diseases and the types of models used in the study. Our GSEA results showed that EA at specific stimulation mode could activate autophagy (Figure 7) and inhibit TORC2 signal (GO: 0038203, *P* = 0.0311), and the genes involved were significantly down-regulated Prr5l (Table 4). Therefore, we believe that autophagy is closely related to the translocation and degradation of TJ proteins in BBB, which is also an idea to study the mechanism of EA opening BBB. However, the protein expression and mRNA expression are not completely consistent, because the sample of this experiment is the total RNA of rat cortical tissue, not a single cell, which is also one of the limitations of this experiment. Future studies should first confirm whether the autophagy pathway activated by EA is involved in the translocation and degradation of BBB TJ protein. Secondly, whether the degree of autophagy activation is enough to cause cell damage. Finally, is the autophagy activation after EA a direct effect or is it because EA changes the cellular distribution of TJ proteins (such as translocation from cell membrane to cytoplasm), thus promoting autophagy activation.

In addition, EA may have an effect on the clathrin-coated pit (GO:0005905, *P* = 0.0424), involving genes including *Cttn* and *Sh3bp4* (Table 4). Clathrin-coated pit is a part of the endomembrane system in the form of an invagination of a membrane upon which a clathrin coat forms, and that can be converted by vesicle budding into a clathrin-coated vesicle. Coated pits form on the plasma membrane, where they are involved in receptor-mediated selective transport of many proteins and other macromolecules across the cell membrane. Experimental studies on various animal models have shown that Aβ is mainly cleared through the BBB across blood vessels (70–85%), while a small part is cleared through the ISF flow (Bading et al., 2002; Deane et al., 2004; Tarasoff-Conway et al., 2015) and the molecular mechanism of Aβ clearance through BBB has been clarified in more detail recently. In short, Aβ produced in the brain binds to LRP1 on the albumin side of BBB, resulting in its rapid internalization into endothelial cells and clearance through blood. Phosphatidylinositol-binding clathrin assembly protein (PICALM) is crucial for the internalization of LRP1 – Aβ complex mediated by endothelial clathrin/PICALM, and guides the transport of endocytosis vesicles containing Aβ in endothelial cells by sequential fusion with Rab5 positive early endosomes and Rab11 positive sorting endosomes used for the endocytosis of BBB luminal (Zhao et al., 2015).

However, as we mentioned above, our sample is a mixture of all cells in the cortex, which can not directly prove that these genes and proteins are expressed in which kind of cells of the blood-brain barrier. Our research mainly focuses on the role of endothelial cells, and the blood-brain barrier is not a single entity. It is composed of astrocytes, pericytes and endothelial cells, which allow the endothelial cells of the central nervous system to strictly regulate the movement of ions, molecules and cells between blood and neural tissues. Astrocytes are the most abundant cells in the brain, which are very important in regulating brain and BBB function. A recent breakthrough study (Kitchen et al., 2020) proved that targeting astrocytes at the blood-brain barrier (BBB) and spinal cord barrier (BSCB) is a feasible option for the treatment of TBI and spinal cord injury. This study showed that pharmacological inhibition of these signal events could prevent the development of central nervous system edema and promote the functional recovery of injured rats, which was confirmed by Sylvain et al. (2021). Recently, the molecular mechanism of clearing Aβ through BBB has been elucidated in more detail. Lymphatic pathway is a waste removal system, which plays an important role in dementia. It uses the unique system of perivascular channels to promote the effective removal of soluble proteins and metabolites from the central nervous system. AQP4 is highly expressed in astrocytes. The enrichment of perivascular AQP4 at BSCB/BBB shows that it plays a role in lymphatic function. The development of new drugs for this system will have great therapeutic potential (Salman et al., 2021). In addition, pericytes also play an important role in the maintenance of BBB function. In addition to communicating with astrocytes, pericytes also support the maintenance of BBB in postnatal brain. Pericyte deficient mutant mice showed increased permeability of BBB to low molecular weight and high molecular weight tracers, and pericytes can directly regulate the expression of transporters (Arvanitis et al., 2020).

Achieving adequate BBB transmission is a key challenge in the development of drugs for the treatment of central nervous system (CNS) diseases. However, most traditional BBB opening strategies are difficult to be applied in clinic because of their extensive and non-specific regulation of BBB, resulting in damage to normal brain tissue. Therefore, non-invasive methods that selectively and effectively cross the BBB, such as receptor-mediated cell transport and the use of neurophilic viruses, nanoparticles and exosomes, have become popular in recent years (Terstappen et al., 2021). For example, in the process of glioma treatment, the poor permeability of BBB is the main reason that restricts the application of newly developed treatment methods for glioma in clinic. Researchers have proposed a variety of strategies to target and effectively regulate the BBB of tumor, so as to obtain more effective glioma treatment (Luo and Shusta, 2020).

## Conclusion

Our research will provide valuable resources for discovering and studying new targets and signaling pathways that mediate crosstalk between different cell types in NVU. This may lead to the development of new transgenic animal models – BBB, NVU, and pluripotent stem cell models of different neurological diseases, as a valuable way to discover and test new drug delivery methods.

## Data Availability Statement

The original contributions presented in the study are publicly available. This data can be found here: https://www.ncbi.nlm.nih.gov/geo/, GSE192885.

## Ethics Statement

This study was approved by the Animal Protection and Use Committee of Zhejiang University of Traditional Chinese Medicine.

## Author Contributions

CM was responsible for drafting and writing the manuscript. LG and HW analyzed and sorted out the data. LR and YL provided help for the experiment. SZ and PG contributed to make important modifications to the manuscript. XL approved the final manuscript to be published. All authors contributed to the article and approved the submitted version.

## Conflict of Interest

The authors declare that the research was conducted in the absence of any commercial or financial relationships that could be construed as a potential conflict of interest.

## Publisher’s Note

All claims expressed in this article are solely those of the authors and do not necessarily represent those of their affiliated organizations, or those of the publisher, the editors and the reviewers. Any product that may be evaluated in this article, or claim that may be made by its manufacturer, is not guaranteed or endorsed by the publisher.

## Footnotes

1
https://cutadapt.readthedocs.io/en/stable/

2
https://daehwankimlab.github.io/hisat2/

3
http://ccb.jhu.edu/software/stringtie/

4
http://ccb.jhu.edu/software/stringtie/gffcompare.shtml

5
http://www.bioconductor.org/packages/release/bioc/html/ballgown.html

6
http://www.bioconductor.org/packages/release/bioc/html/DESeq2.html

7
http://metascape.org

8
http://string-db.org/
